# Bronchial sleeve anastomosis in children: a single-center experience demonstrating safety and efficacy

**DOI:** 10.3389/fped.2026.1740253

**Published:** 2026-02-02

**Authors:** Min Da, Tao Wang, Yang Wang, Jiexin Yu, Yang Xu, Xuming Mo, Jirong Qi

**Affiliations:** 1 Department of Cardiothoracic Surgery, Children’s Hospital of Nanjing Medical University, Nanjing, China; 2 Department of Cardiovascular Surgery, Qinghai University Affiliated Hospital, Xining, China

**Keywords:** anastomotic complications, bronchial sleeve anastomosis, children, trauma, tumor

## Abstract

**Objective:**

To evaluate the technical feasibility, safety, and clinical outcomes of bronchial sleeve anastomosis in a pediatric case series encompassing two main etiologies (trauma and tumor).

**Methods:**

A retrospective analysis was conducted on 8 pediatric patients who underwent bronchial sleeve resection at our center between May 2018 and May 2025. Preoperative diagnosis was confirmed by computed tomography, magnetic resonance imaging, and bronchoscopy. Collected data included demographic characteristics, surgical parameters, pathological results, perioperative outcomes, and follow-up information.

**Results:**

All eight procedures were successfully completed without perioperative mortality. The mean operative time was 306.88 ± 127.31 min, with mean intraoperative blood loss of 51.25 ± 23.57 mL. The mean duration of mechanical ventilation was 16.06 ± 12.57 h, chest tube drainage was maintained for 234.86 ± 91.04 h, and the mean postoperative hospital stay was 25.00 ± 8.45 days. The median follow-up period was 2.7 years (range: 0.6–7.1 years). Perioperative complications included two cases of mild anastomotic stenosis, both successfully managed with bronchoscopic cryotherapy, and one case of chylothorax that resolved with conservative drainage. The trauma group required significantly longer postoperative mechanical ventilation compared to the tumor group (*P* < 0.05). At the last follow-up, all patients were alive with patent airways, recovered pulmonary function, and had resumed normal activities. No tumor recurrence was observed in the oncology patients.

**Conclusion:**

Bronchial sleeve resection represents a safe, feasible, and effective lung-preserving procedure in carefully selected pediatric patients. This technique allows complete lesion removal while maximizing pulmonary function preservation and promoting long-term quality of life, establishing it as a preferred surgical option for children with severe airway trauma or bronchial tumors.

## Introduction

Bronchial sleeve anastomosis is a lung-preserving surgical procedure that involves resection of the diseased bronchial segment followed by end-to-end anastomosis to reconstruct airway continuity, thereby avoiding pneumonectomy or lobectomy (1, 2). Compared with pulmonary resection, this technique has demonstrated superior short- and long-term outcomes in cancer patients, as supported by multiple clinical studies (3–5).

In pediatric patients, preserving functional lung tissue is particularly crucial during critical stages of growth and development, as it significantly impacts long-term respiratory function and quality of life (6). However, the smaller bronchial diameter and more fragile tissue structure in children impose higher demands on surgical precision and perioperative management (7). To date, only case reports and small case series have suggested that bronchial sleeve resection and reconstruction can achieve favorable outcomes in pediatric patients when strict indications are applied (7, 8). Nevertheless, the safety and efficacy of this procedure in children require further validation.

This study presents a retrospective, descriptive case series of eight pediatric patients who underwent bronchial sleeve anastomosis at our center for either traumatic bronchial rupture or bronchial tumors. We aimed to describe the perioperative management, evaluate the technical feasibility and safety of this procedure, and report the clinical outcomes in this heterogeneous pediatric cohort.

## Materials and methods

### Patients and design

This study included eight pediatric patients who underwent bronchial segment resection with sleeve end-to-end anastomosis between May 2018 and May 2025 in our department. Preoperative localization of the lesion was confirmed using computed tomography (CT), magnetic resonance imaging (MRI), and ultrasonography. Clinical data were retrospectively collected from the hospital information system, including: 1) Baseline characteristics: name, sex, age, height, weight, and body mass index (BMI); 2) Disease-related information: symptoms and signs, pathological diagnosis, laboratory findings, and imaging results; 3) Surgical and postoperative recovery data: surgical approach, operative duration, intraoperative blood loss, postoperative hospital stay, complications, and follow-up status after discharge. Patients were carefully selected for this parenchyma-sparing procedure based on comprehensive assessment of anatomical resectability, functional viability of the distal lung, and feasibility of tension-free anastomosis. Specifically, for cases with delayed presentation and lung collapse, preoperative functional assessment involved contrast-enhanced CT to evaluate vascular patency and bronchoscopy to inspect the airway.

The study was conducted in accordance with the ethical principles of the World Medical Association's Declaration of Helsinki for medical research involving human subjects (9). The study protocol was approved by the Ethics Committee of the Children’s Hospital of Nanjing Medical University (No. 202510011-1).

### Surgical techniques

All surgical procedures were performed by the same team of three experienced thoracic surgeons. Under general endotracheal intubation, optimal exposure was achieved by selecting double-lumen intubation, bronchial blockade, or single-lung ventilation on the unaffected side according to the lesion location. A posterolateral thoracotomy was routinely adopted to fully mobilize and expose the affected bronchus.

**Figure 1:**
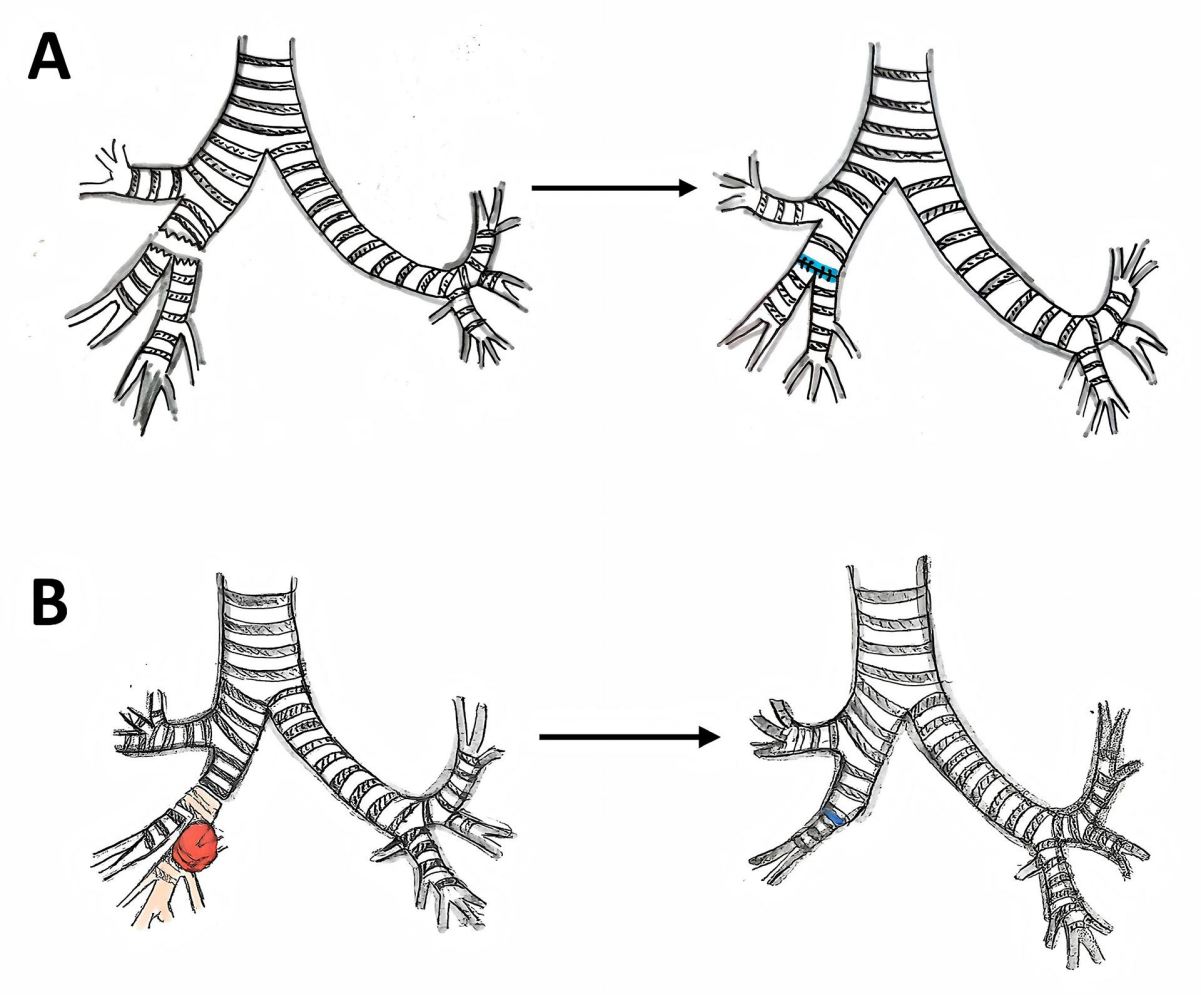
Schematic diagram of the surgical procedures: **(A)** procedure for trauma case 5; **(B)** procedure for tumor case 3.

Specific management strategies were tailored to the underlying etiology: in cases of traumatic bronchial rupture, surrounding hematoma and granulation tissue were debrided, and the stump was trimmed to healthy airway tissue; for tumor patients, the bronchus was transected at least 0.5–1.0 cm beyond the proximal and distal margins of the tumor to ensure a negative resection margin, which was confirmed by intraoperative frozen section analysis. In instances where the tumor invaded the adjacent pulmonary lobe, a combined lobar sleeve resection was performed, and airway continuity was reestablished via end-to-end anastomosis using 5-0 PDS absorbable monofilament sutures (Figure 1).

The anastomotic technique strictly adhered to three core principles: tension reduction, preservation of blood supply, and negative margins. Tension was minimized through a continuous suture of the membranous portion combined with interrupted sutures of the cartilaginous portion, supplemented when necessary by hilar or mediastinal release. To mitigate the risk of anastomotic leakage and hemorrhage, the anastomosis was wrapped with an intercostal muscle flap or pleural soft tissue. Intraoperative frozen section analysis was conducted in all tumor cases, with the extent of lymph node sampling determined according to pathological findings (10). Bronchoscopy was routinely performed upon completion of the procedure to verify airway patency and satisfactory suturing, while an air leak test conducted by the anesthesiologist confirmed the absence of air leakage and assessed lung re-expansion.

### Follow-up

Patients were followed up through outpatient visits and telephone interviews. Regular follow-ups were scheduled at 1, 3, 6, and 12 months postoperatively, and subsequently extended to every six months or annually. Evaluation parameters included clinical symptoms (cough, wheezing, and exercise tolerance), physical signs, and imaging studies. Bronchoscopy was performed when clinically indicated to assess mucosal healing, granulation tissue formation, and the risk of stenosis. For pediatric tumor cases, additional surveillance for disease recurrence was conducted.

## Results

### Baseline patient characteristics

A total of 8 pediatric patients were included in this study, comprising 5 trauma cases and 3 tumor cases. Among the trauma patients, 1 was male and 4 were female; 3 involved the left bronchus and 2 the right bronchus. All tumor patients were male, with 2 involving the left side and 1 the right side. The mean age of the cohort was 91.25 ± 36.62 months (Table 1).

**Table 1:** General characteristics of the patients.

Variables	Total	Classification		*P* Value
		Traumatic cases	Tumor cases	
	(*n* = 8)	(*n* = 5)	(*n* = 3)	
Sex, *n* (%)				0.14
Female	4 (50.00)	4 (80.00)	0 (0.00)	
Male	4 (50.00)	1 (20.00)	3 (100.00)	
Age(months)	106.00 (64.50, 112.50)	71.00 (45.00, 123.00)	106.00 (106.00, 107.50)	0.76
Height (cm)	140.00 (111.75, 149.00)	115.00 (102.00, 155.00)	145.00 (140.00, 146.00)	0.79
Weight (Kg)	31.00 (20.75, 36.62)	22.00 (17.00, 36.50)	35.00 (31.00, 36.00)	0.57
BMI	16.48 (15.52, 16.64)	16.34 (15.62, 16.62)	16.65 (15.73, 16.88)	0.57
Site of lesion, *n* (%)				1.00
Left	5 (62.50)	3 (60.00)	2 (66.67)	
Right	3 (37.50)	2 (40.00)	1 (33.33)	

Traumatic bronchial rupture in all 5 cases resulted from blunt injury, such as traffic accidents or falls from height. The tumor group consisted of one case each of mucoepidermoid carcinoma, inflammatory myofibroblastic tumor, and neuroendocrine tumor.

All patients presented with preoperative respiratory symptoms. The trauma group exhibited acute manifestations, including dyspnea, mediastinal emphysema, or tension pneumothorax, with imaging primarily revealing pulmonary atelectasis. In contrast, the tumor group had an insidious onset, with most patients presenting due to recurrent atelectasis or infection; computed tomography (CT) identified bronchial occupying lesions in these cases.

The interval from injury to surgery in trauma cases ranged from 14 h to 29 days. Tumor diameter varied from 2 to 7 cm. All tumor cases were confirmed by preoperative bronchoscopic biopsy (Tables 2, 3, and Supplementary S1).

**Table 2:** Demographic data and clinical characteristics of patients with trauma.

Case	Sex	Age (months)	Affected Site	Interval from Injury to Surgery (days)	Follow-up (months)	Status
1	Female	123	Left main bronchus	15	13	Alive
2	Female	135	Left main bronchus	29	6	Alive
3	Male	35	Right main bronchus	0.5	76	Alive
4	Female	71	Left main bronchus	28	28	Alive
5	Female	45	Bronchus intermedius, right middle and lower lobe bronchi	2.5	3	Alive

**Table 3:** Demographic data and clinical characteristics of patients with tumor.

Case	Sex	Age (months)	Affected Site	Pathology	Tumor Size (cm)	Follow-up (months)	Status
1	Male	109	Left main bronchus	Mucoepidermoid carcinoma	2	44	Alive
2	Male	106	Left main bronchus	Inflammatory myofibroblastic tumor	4	6	Alive
3	Male	106	Bronchus intermedius and right lower lobe bronchus	Neuroendocrine tumor	7	6	Alive

### Surgical and postoperative results

All eight pediatric patients successfully underwent bronchial sleeve anastomosis. In the trauma group, lesions involved the left main bronchus (*n* = 3), right main bronchus (*n* = 1), and bronchus intermedius (*n* = 1). All traumatic cases were managed with debridement and end-to-end anastomosis, without lobectomy. In the tumor group, lesions were located in the left main bronchus (*n* = 2) and right lower lobe bronchus (*n* = 1). Two patients underwent bronchial sleeve resection alone, while one patient required combined sleeve resection of the right lower lobe due to tumor involvement of the lobar orifice. All tumor patients achieved R0 resection, confirmed by intraoperative frozen section. Systematic lymph node dissection or sampling was performed based on preoperative and intraoperative findings, with systematic lymph node dissection ultimately conducted in all cases; one tumor patient (Case 3) had lymph node metastasis.

Postoperatively, all patients were transferred to the intensive care unit. The mean extubation time was 16.06 ± 12.57 h, chest tube duration was 234.86 ± 91.04 h, and postoperative hospital stay was 25.00 ± 8.45 days. The trauma group required significantly longer postoperative mechanical ventilation than the tumor group (*P* < 0.05), attributable to associated pulmonary contusion and other injuries. No other significant intergroup differences were observed (Table 4). It is worth noting that in clinical practice, compared with relatively simple selective airway reconstruction surgeries, pediatric tumor surgeries typically require longer operation times and recovery periods, even though these differences do not reach a statistically significant level (*P* > 0.05).

**Table 4:** Surgery and postoperative conditions.

Variables	Total	Classification		***P* Value**
		Traumatic cases	Tumor cases	
	(*n* = 8)	(*n* = 5)	(*n* = 3)	
Surgical operation time (min)	300.00 (207.50, 348.75)	215.00 (185.00, 315.00)	345.00 (315.00, 457.50)	0.25
Intraoperative blood loss (mL)	50.00 (45.00, 52.50)	50.00 (30.00, 50.00)	60.00 (55.00, 80.00)	0.06
Ventilator usage time (h)	15.00 (8.75, 20.12)	20.00 (18.00, 20.50)	2.00 (1.50, 6.50)	0.04
Intensive care unit time (h)	176.50 (138.75, 202.25)	161.00 (155.00, 201.00)	192.00 (141.00, 199.00)	1.00
Thoracic tube drainage time (h)	200.00 (150.75, 263.75)	209.00 (155.00, 251.00)	191.00 (164.50, 246.50)	1.00
Inpatient after surgery (d)	28.50 (18.50, 29.25)	28.00 (20.00, 29.00)	29.00 (21.50, 29.50)	0.76

Two patients developed mild anastomotic stenosis, which improved after bronchoscopic cryotherapy without restenosis during follow-up. One case of chylothorax resolved completely with adequate drainage. There were no perioperative deaths. Histopathology confirmed mucoepidermoid carcinoma (ICD-O: 8430/3) in Tumor Case 1, who received 3 months of nab-paclitaxel plus carboplatin chemotherapy followed by regular outpatient surveillance. Tumor Case 2 was diagnosed with inflammatory myofibroblastic tumor with ALK rearrangement and no nodal metastasis. Tumor Case 3 had a neuroendocrine tumor (atypical carcinoid, G2) with metastasis in two lymph node stations, classified as pathological stage T1cN2bM0 (IIIA). Postoperative PET-CT showed no residual disease, and the patient continues regular outpatient follow-up.

### Postoperative follow-up results

The median postoperative follow-up was 2.74 years (range: 0.6–7 years). Three-month postoperative CT reconstructions in all patients demonstrated satisfactory bronchial growth without luminal narrowing due to anastomotic scarring or indications for reintervention. Four patients underwent bronchoscopy, which further confirmed excellent anastomotic healing and adaptation to pediatric airway growth. At the last follow-up, all patients were alive with well-recovered respiratory function, free from long-term mortality or tumor recurrence, and had resumed age-appropriate daily and physical activities.

### Typical case

**Figure 2:**
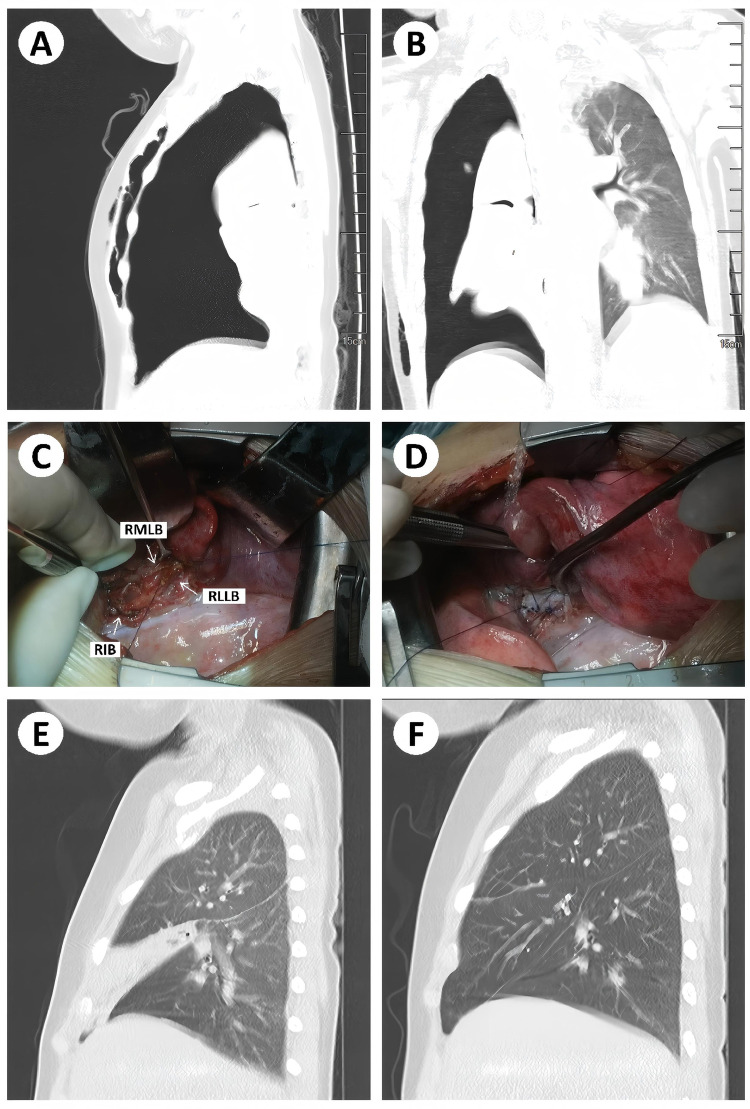
Traumatic case 5, a 3-year-old girl admitted following a traffic accident with coma. Surgery was performed after stabilization with closed thoracic drainage. **(A,B)** Preoperative CT images demonstrating right massive pneumothorax, pulmonary atelectasis, and an obscured right middle lobe bronchus. **(C,D)** Intraoperative findings revealed a fragmented transection of the distal bronchus intermedius, followed by mobilization and anastomosis of the right middle and lower lobe bronchi. Intraoperative bronchoscopy confirmed anastomotic patency. **(E)** CT at 2 weeks postoperatively showed atelectasis of the right middle lobe; bronchoscopy identified anastomotic granulation tissue, which was treated with local cryotherapy. **(F)** Three-month postoperative CT indicated restored bronchial lumen and satisfactory re-expansion of the middle lobe.

A 3-year-old girl was admitted following a traffic accident with coma. CT revealed right massive pneumothorax, atelectasis, and an obscured right middle lobe bronchus. After stabilization with thoracic drainage, surgery identified a fragmented transection of the distal right intermediate bronchus. The right middle and lower lobe bronchi were mobilized and anastomosed. Intraoperative bronchoscopy confirmed anastomotic patency. Postoperative bronchoscopy detected anastomotic granulation tissue, which resolved after cryotherapy (Figure 2).

**Figure 3:**
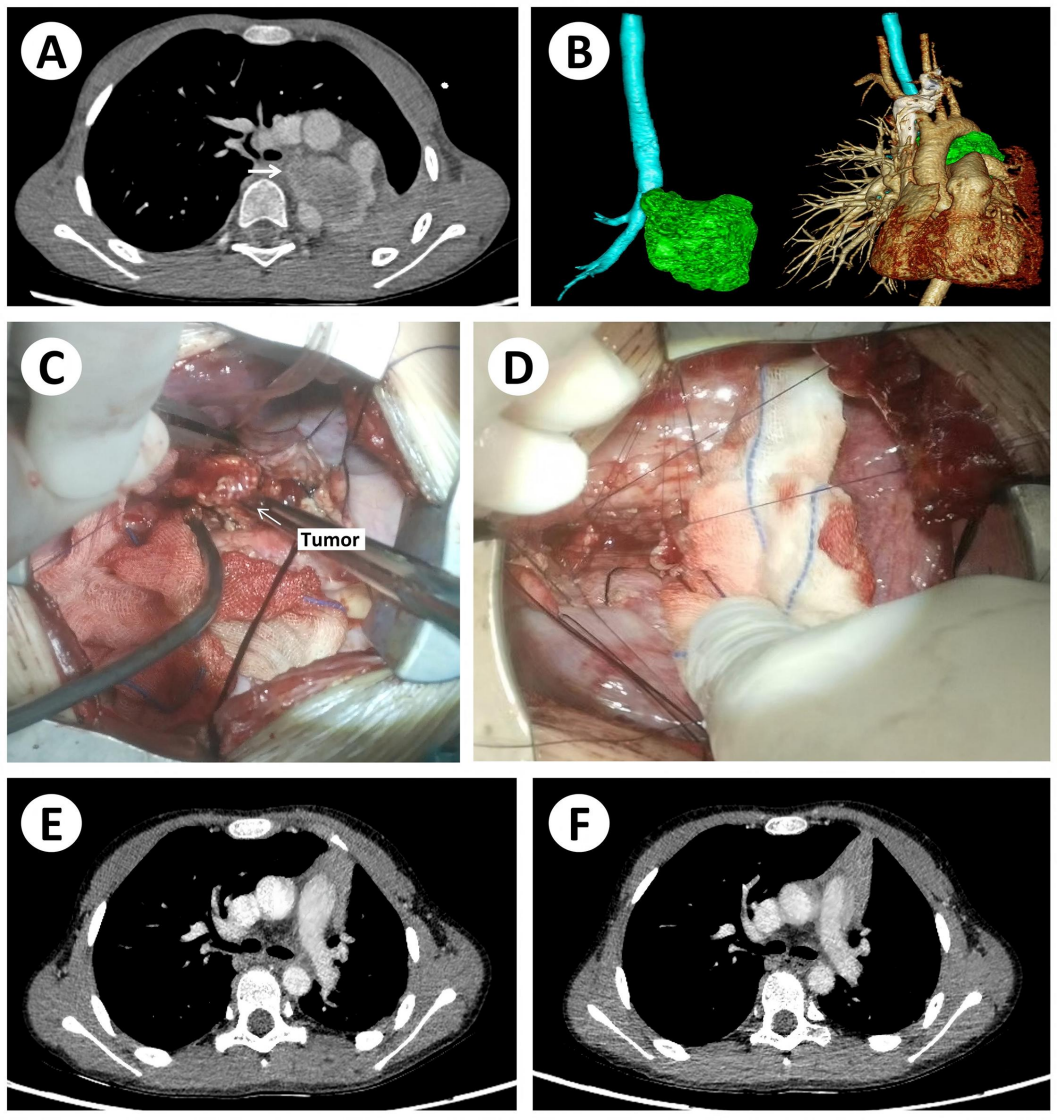
Tumor case 2, an 8-year-old boy presenting with cough. CT suggested a suspected tumor. Bronchoscopic biopsy confirmed the diagnosis of inflammatory myofibroblastic tumor, and FISH revealed ALK rearrangement. **(A,B)** Preoperative CT and three-dimensional reconstruction. **(C)** Intraoperative view showing tumor dissection and exposure. **(D)** Bronchial anastomosis following tumor resection. **(E,F)** Postoperative CT scans at 1 month and 4 months, respectively. After one month of crizotinib targeted therapy, CT showed tumor reduction, but the child developed significant dyspnea. Multidisciplinary team evaluation indicated a high likelihood of achieving surgical T0 resection. The patient subsequently underwent complete tumor resection with sleeve anastomosis of the left main bronchus, accompanied by mediastinal and hilar lymph node sampling. Continued postoperative targeted therapy was administered, and follow-up showed no recurrence.

An 8-year-old boy presented with a persistent cough. CT suggested a bronchial tumor, and bronchoscopic biopsy confirmed inflammatory myofibroblastic tumor with ALK rearrangement by FISH. After one month of crizotinib therapy, CT showed tumor regression. The patient subsequently underwent complete tumor resection with sleeve anastomosis of the left main bronchus and lymph node dissection. Continued targeted therapy and follow-up have shown no recurrence (Figure 3).

## Discussion

In this series, all eight pediatric patients successfully underwent bronchial sleeve anastomosis without perioperative mortality, achieving satisfactory postoperative airway patency and pulmonary function recovery. These outcomes demonstrate that, with strict patient selection, thorough preoperative preparation, and an experienced surgical team, this procedure is both safe and feasible in children. Our selection criteria primarily involved assessing anatomical resectability, confirming the functional salvageability of the distal lung parenchyma, and ensuring the technical feasibility of a tension-free anastomosis. Based on our institutional experience, the primary indications for pediatric bronchial sleeve anastomosis include: severe airway trauma (main bronchus transection), central benign bronchial tumors (such as carcinoid, mucoepidermoid carcinoma, and inflammatory myofibroblastic tumor), localized low-grade malignancies, and selected complex bronchial stenoses.

Traumatic and neoplastic etiologies differ in surgical decision-making and prognosis. For traumatic bronchial rupture, surgical timing and strategy depend on injury severity and diagnostic timeliness (2, 11). Acute-phase repair can promptly restore ventilation and prevent pulmonary collapse or recurrent infection. Patients requiring delayed surgery due to unstable vital signs from associated injuries often present with scarring, infection, and adhesions, complicating the procedure; nonetheless, even after resection of the stenotic segment, end-to-end anastomosis can yield excellent functional recovery. All five trauma patients in our cohort underwent bronchial sleeve anastomosis after diagnosis. Two patients with unstable vital signs due to combined injuries and hemopneumothorax underwent initial thoracic drainage, followed by surgery after stabilization, with the longest interval from injury to surgery being 29 days. Postoperative imaging confirmed satisfactory lung re-expansion in all cases.

Neoplastic bronchial lesions, in contrast, are typically addressed electively. Most pediatric bronchial tumors are benign or low-grade malignant, with rare distant metastasis (12–14). Surgery should prioritize lung parenchyma preservation while achieving complete resection. Compared to traditional lobectomy or pneumonectomy, bronchial sleeve anastomosis allows for local resection of the involved bronchial segment with airway reconstruction, thereby avoiding significant pulmonary function loss (4, 15–17). Key intraoperative considerations include complete tumor excision with negative margins and systematic (or sampling) lymph node evaluation for accurate staging. Although tumor surgery is elective and offers better control over perioperative factors—leading to a lower incidence of early complications—preoperative atelectasis and recurrent infection due to bronchial obstruction necessitate focused attention on lung re-expansion and infection control postoperatively (18, 19). In our cohort, all three tumor patients showed no recurrence during follow-up and exhibited excellent lung re-expansion, consistent with previously reported high cure rates and long-term survival following sleeve resection for low-grade malignancies in children (20). Overall, while bronchial sleeve anastomosis for traumatic vs. neoplastic indications differs in urgency, surgical strategy, and follow-up priorities, both can achieve favorable outcomes when adhering to standardized oncological and reconstructive principles.

All patients in this series underwent preoperative CT and fiberoptic bronchoscopy, establishing a standardized assessment protocol incorporating three-dimensional CT reconstruction and bronchoscopy (3, 21). Bronchoscopy provided definitive diagnosis in all cases, with pathological confirmation via biopsy in tumor patients, thereby informing subsequent treatment planning. Single-lung ventilation strategies (double-lumen intubation or bronchial blockade) were collaboratively designed by anesthesiology and thoracic surgery teams preoperatively and adjusted intraoperatively as needed (22). In one tumor case involving the left main bronchus, single-lung ventilation was successfully achieved using a right-sided cuffed endotracheal tube.

Postoperative management focused on promoting lung re-expansion and functional recovery, including adequate analgesia, respiratory physiotherapy, nebulization, and early chest tube removal (23, 24). The trauma group required significantly longer postoperative mechanical ventilation than the tumor group (22.3 ± 11.0 h vs. 5.7 ± 7.2 h, *P* = 0.04), consistent with the higher physiological burden of pulmonary contusion, contamination, and inflammatory load in trauma patients. Conversely, it is important to note that tumor surgery in children can also be complex due to factors such as tumor size, location, and the requirement for complete oncologic resection. These complexities may extend operative times and influence recovery parameters compared to simpler elective reconstructions. Furthermore, in cases of intraoperative poor lung re-expansion, advanced techniques such as selective pulmonary venous blood gas analysis can guide real-time preservation decisions. For instance, maintaining a PaO_2_ > 60 mmHg during low-FiO_2_ ventilation may justify lobe retention despite suboptimal expansion, underscoring the principle of maximizing parenchymal salvage in pediatric surgery (25).

Anastomotic complications (stenosis, dehiscence or fistula) are primarily associated with ischemia and tension at the anastomotic site, with inflammatory burden being a significant contributing factor (26). Accordingly, we adopted a strategy centered on “tension reduction–preservation of blood supply–soft tissue coverage–protocolized endoscopic follow-up” employing minimally invasive endoscopic reintervention (cryotherapy or balloon dilation) as first-line treatment. Two patients who developed anastomotic granulation tissue regained airway patency after cryotherapy, and there were no serious complications such as bronchopleural fistula. This supports the feasibility and safety of this management approach.

This study has several limitations. First, as a single-center retrospective analysis, case selection was inevitably subject to potential bias. Most importantly, the small overall sample size (*n* = 8) and the heterogeneous distribution of etiologies (trauma vs. tumor) render this study underpowered to conduct valid comparative statistical analyses. Therefore, any between-group comparisons presented should be interpreted strictly as descriptive observations. Furthermore, although the median follow-up period covered the crucial postoperative recovery phase, extended surveillance is needed to assess long-term outcomes. This is particularly true for tumor patients, especially those with low-grade malignancies, in order to monitor for late effects and potential delayed complications. Future multi-center collaborations, larger sample sizes, and long-term prospective studies are warranted to further validate the selection criteria and therapeutic efficacy of bronchial sleeve anastomosis in children.

## Conclusion

This single-center retrospective study confirms that bronchial sleeve anastomosis is a safe, feasible, and effective treatment strategy for carefully selected pediatric patients. The principal advantage of this technique lies in its ability to thoroughly remove the lesion while maximally preserving healthy lung parenchyma, thereby providing essential support for long-term pulmonary function. Whether applied in emergency settings to salvage lung tissue after traumatic bronchial rupture or electively for tumor resection with curative intent, the procedure demonstrates favorable short- and mid-term clinical outcomes with an acceptable complication profile. Based on our findings, we recommend bronchial sleeve anastomosis as a preferred surgical option for pediatric airway diseases meeting the appropriate indications in experienced centers. However, this study has inherent limitations, including its single-center design, small sample size, etiological heterogeneity, and a follow-up duration insufficient for long-term outcome assessment. Consequently, these preliminary findings require confirmation through future well-designed, large-scale multicenter studies. Such research is essential to validate the long-term efficacy, refine perioperative management strategies, and ultimately provide more robust therapeutic options for pediatric patients.

## Data Availability

The original contributions presented in the study are included in the article/Supplementary Material, further inquiries can be directed to the corresponding authors.
